# Supplementary material for: Engineered SUMO/protease system identifies Pdr6 as a bidirectional nuclear transport receptor
Source: J Cell Biol. 2019 Apr 25;218(6):2006–20. doi: 10.1083/jcb.201812091 (PMC6548132; doi:10.1083/jcb.201812091)
Supplement: Supplemental Material (PDF) [file JCB_201812091_sm.pdf]

Supplemental material

Vera Rodriguez et al., https://doi.org/10.1083/jcb.201812091

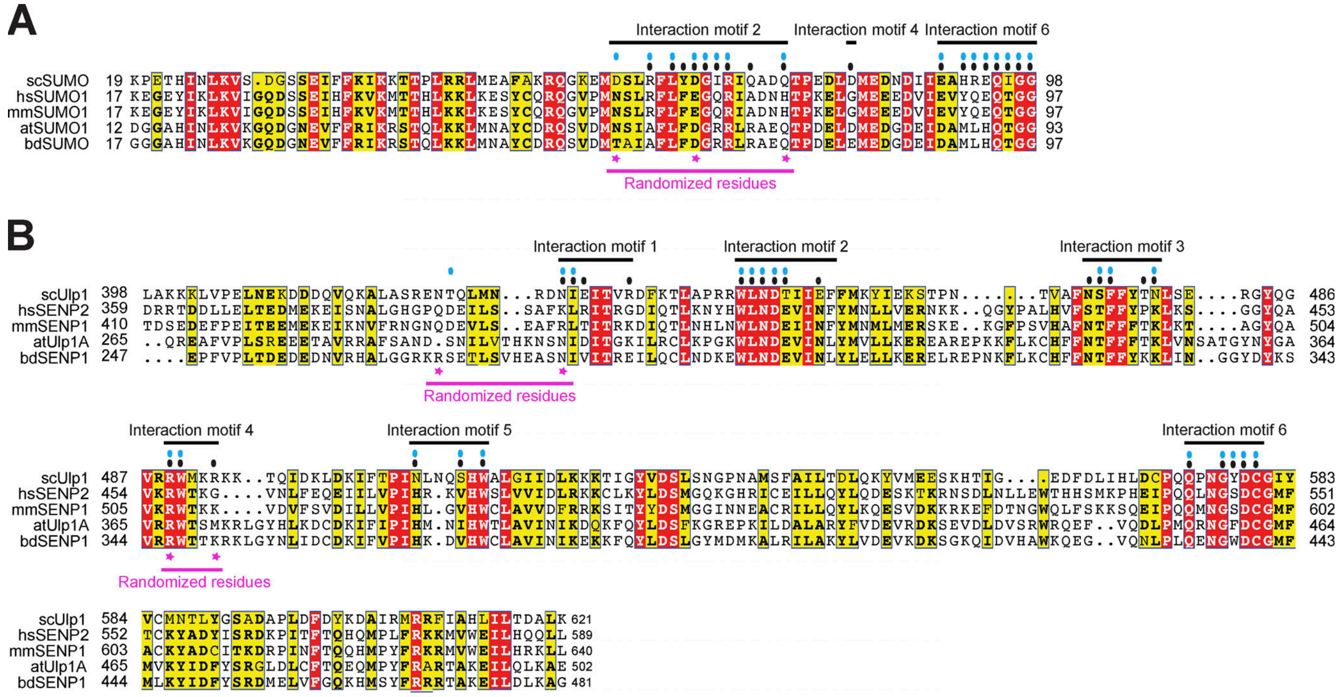

Figure S1. **Mutagenesis strategies for bdSUMO and bdSENP1. (A)** Sequence alignment of SUMO orthologues from *S. cerevisiae* (scSUMO), *Homo sapiens* (hsSUMO1), *Mus musculus* (mmSUMO1), *Arabidopsis thaliana* (atSUMO1), and *Brachypodium distachyon* (bdSUMO). Numberings are according to the respective full-length sequences. Residues of scSUMO that contact its cognate protease scUlp1 are marked by black ovals (PDB 1EUV; Mossessova and Lima, 2000). Residues of hsSUMO1 contacting hsSENP2 are marked by blue ovals (PDB 1TGZ; Reverter and Lima, 2004). Mutagenized residues in bdSUMO are marked by pink stars underneath the aligned sequences. Nomenclature of the interaction motifs is according to Mossessova and Lima (2000). **(B)** Sequence alignment of catalytic domains from various SUMO-protease orthologues. Coloring and numbering of residues are analogous to panel A.

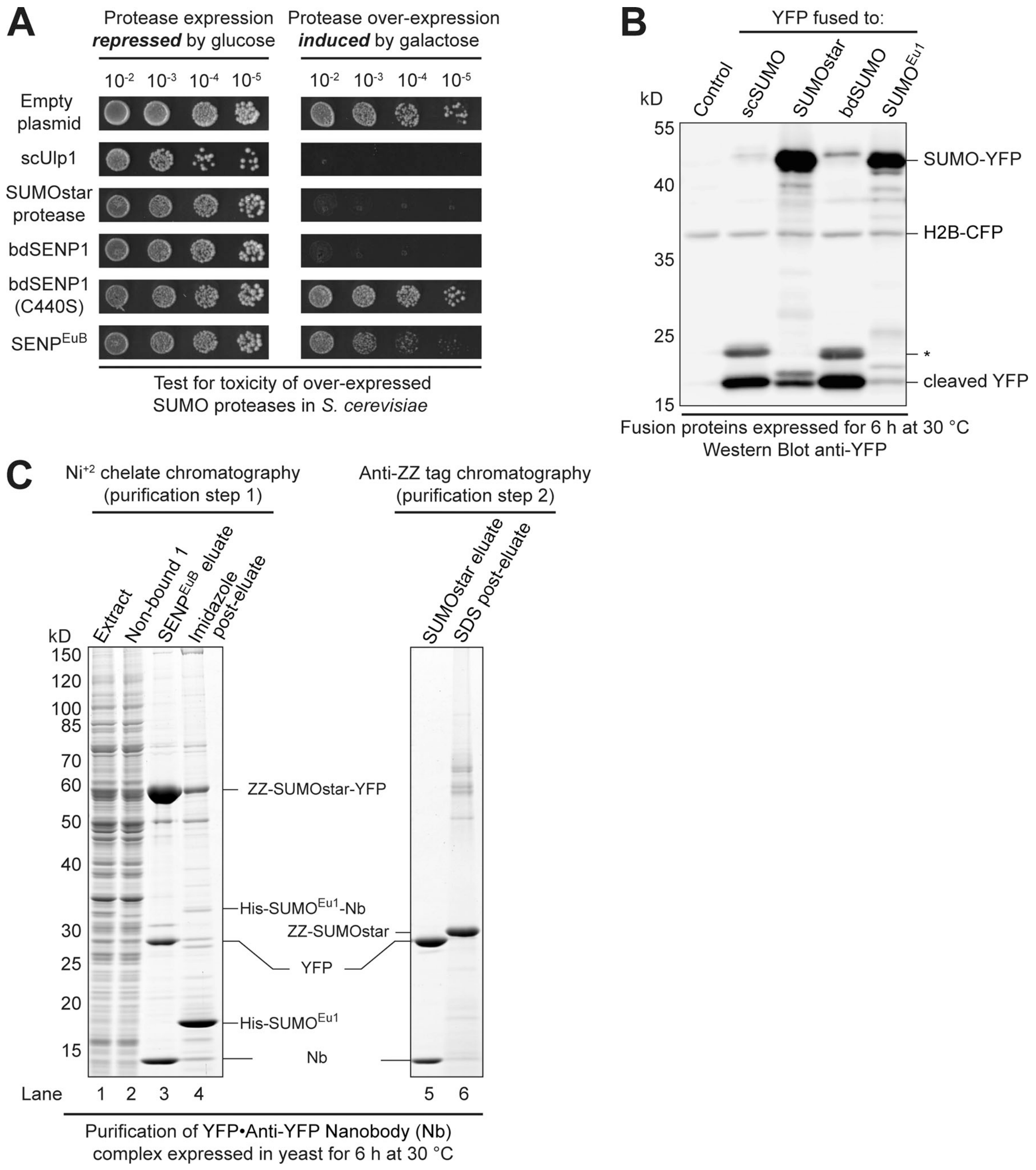

Figure S2. **Applications of the SUMO^{Eu} system in *S. cerevisiae*. (A)** *S. cerevisiae* tolerates overexpression of SENP^{EuB} but not of catalytically active bdSENP1, scUlp1, or SUMOstar protease. Yeast cells were transformed with plasmids encoding the catalytic domain of scUlp1 (residues 403–621), SUMOstar protease, bdSENP1 (residues 242–481), or SENP^{EuB}. An empty vector or a catalytically dead bdSENP1 C440S mutant were used as negative controls. Cells were spotted at indicated dilutions (10^{-2}–10^{-5}) on plates containing glucose or galactose to repress or induce protease overexpression from the Gal4 promoter, respectively. **(B)** SUMO^{Eu1} is a stable tag in *S. cerevisiae*. The stability of overexpressed SUMO–YFP fusions was assessed by blotting with a rabbit anti-YFP antibody, recognizing the fusions, free YFP, the cleaved ZZ-SUMO tags (indicated by an asterisk), and the H2B-CFP loading control. The control strain expressed no SUMO–YFP fusion. SUMO^{Eu1} is by far the most stable fusion partner. **(C)** A heterodimeric complex was formed by coexpressing a ZZ–SUMOstar–YFP fusion with a His-SUMO^{Eu1}-tagged anti-GFP/YFP nanobody in *S. cerevisiae*. An extract was prepared, and His-tagged components were bound to a Ni^{2+}-chelate column and released by SENP^{EuB}-mediated cleavage of the His-tag. The eluate (containing the ZZ–SUMOstar–YFP fusion, free YFP, and the nanobody) was then applied to an anti-ZZ/ED affibody matrix, and specifically bound material was eluted by SUMOstar cleavage, yielding a highly pure YFP-nanobody dimer (lane 5). Panels also show post-eluates from both columns, including the cleaved tags and nonspecifically bound material. Analysis was by SDS-PAGE/Coomassie staining. Load in lanes 1 and 2 corresponds to 1×10^6 cells and load in lanes 3–6 to 3×10^7 cells.

https://doi.org/10.1083/jcb.201812091

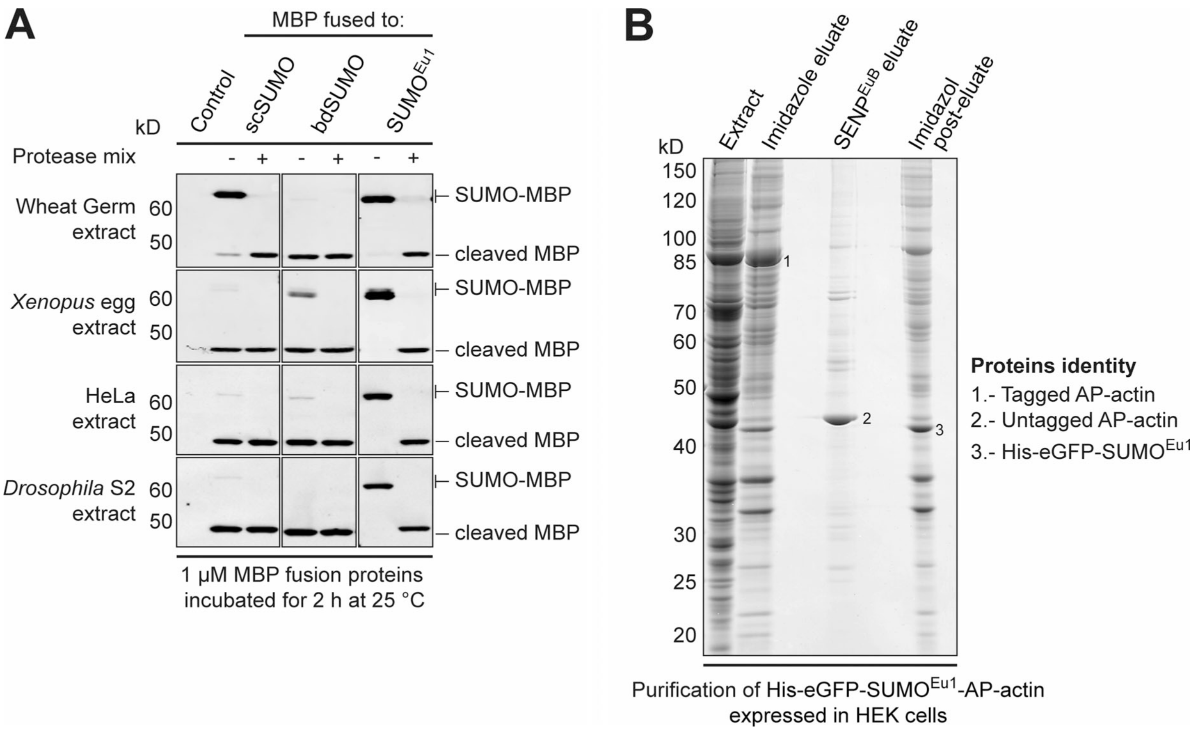

Figure S3. **SUMO^{Eu1} fusions are stable also in plant, frog, human, and insect cytosols. (A)** 1 µM of indicated SUMO–MBP fusions were incubated for 2 h with indicated eukaryotic extracts. Cleavage was then analyzed by anti-MBP blots. All extracts contained sufficient endogenous SUMO protease activity to cut WT–SUMO fusions efficiently. The SUMO^{Eu1} fusion, however, remained fully stable in all extracts and got cleaved only after adding a protease mix comprising 100 nM scUlp1, bdSENP1, and SENP^{EuB} (ruling out inhibitory activities). **(B)** A human nonpolymerizable "AP-actin" mutant (Joel et al., 2004) was expressed with a His-eGFP-SUMO^{Eu1} tag in human HEK-293T cells. An extract was prepared and applied to a Ni^{2+}-chelate matrix. Elution was either with imidazole, which not only desorbed the actin fusion but also abundant (apparently histidine-rich) contaminants. The alternative elution with SENP1^{EuB} released tag-free actin, while the His-eGFP-SUMO^{Eu1} tag and most contaminants appeared in the subsequent post-elution step. Analysis was by SDS-PAGE/Coomassie staining. Load of extract corresponds to roughly 1.5×10^4 cells, load of eluates to $\sim 7.5 \times 10^5$ cells.

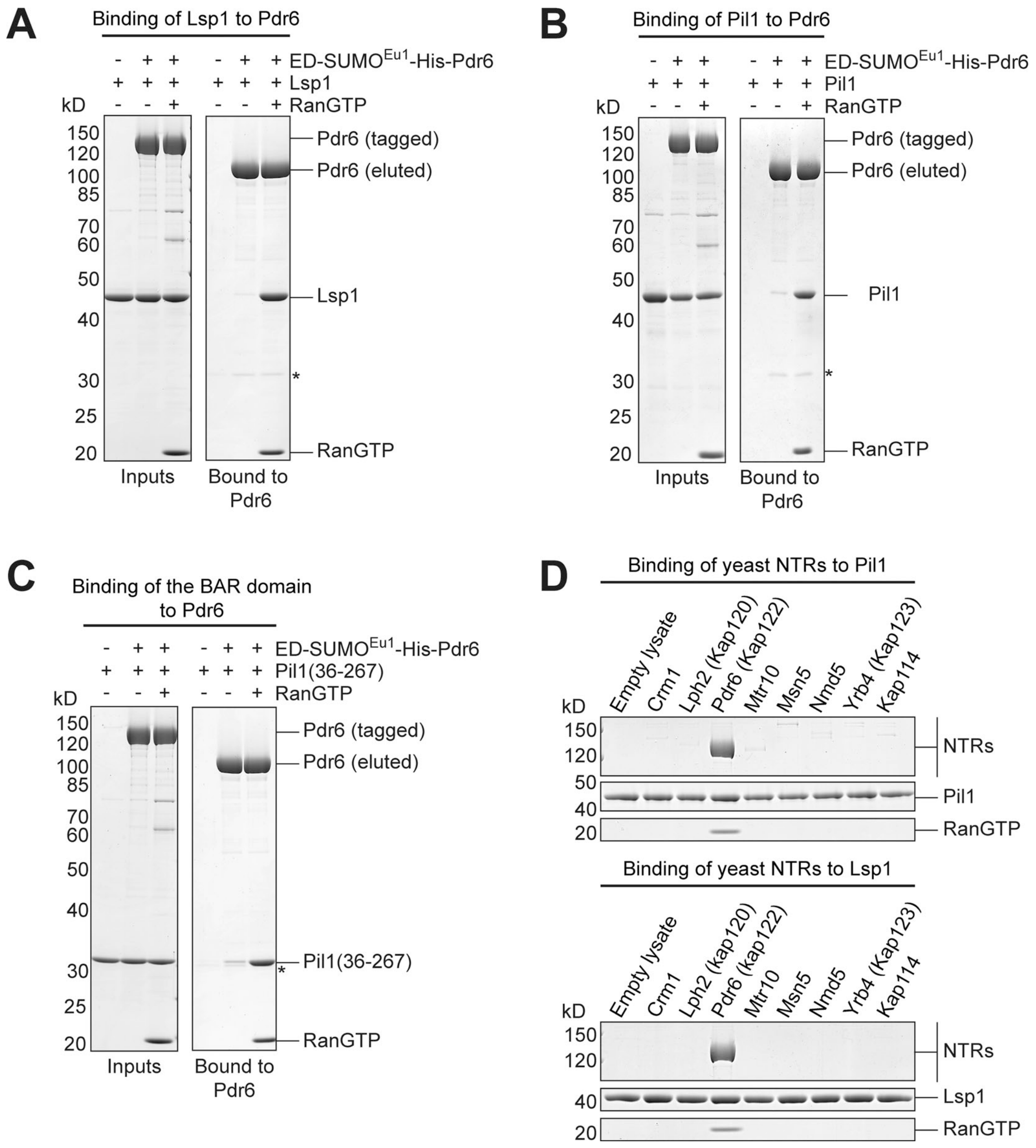

Figure S4. **Pil1 and Lsp1 behave like export cargoes of Pdr6. (A)** ED-SUMO^{Eu1}-His$_{12}$–tagged Pdr6 was mixed with Lsp1 and RanGTP as indicated (Inputs). Formed complexes were retrieved and eluted by NEDP1 (indicated by *). Pdr6 bound Lsp1 in a RanGTP-dependent and thus exportin-like manner. **(B)** The interaction between ED-SUMO^{Eu1}-His$_{12}$–tagged Pdr6 and Pil1 was probed analogously to panel A. Pdr6 bound also Pil1 in an exportin-like manner. **(C)** Assays were as in panel B, but Pil1 was truncated to the boundaries of its BAR domain (residues 36–267). **(D)** H$_{14}$-ZZ-NEDD8–tagged Pil1 and Lsp1 were mixed with *E. coli* lysates containing RanGTP and each a different (potential) yeast exportin. Formed complexes were isolated and analyzed as in B. Note that Pdr6 but no other NTRs was recruited to either Pil1 or Lsp1.

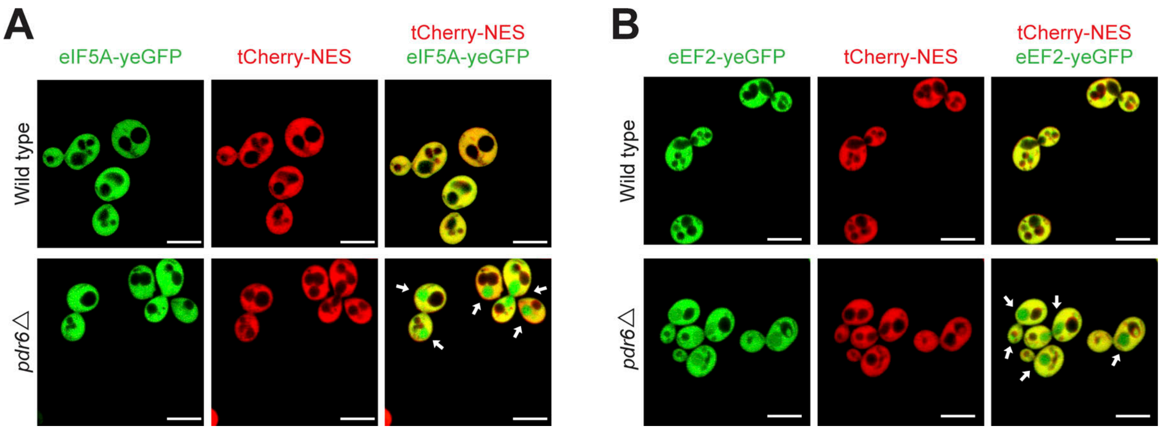

Figure S5. **Localization of eIF5A–eGFP and eEF2–eGFP fusions in WT and *pdr6*Δ cells.** Experiments were performed as in Fig. 6, except that tCherry was fused to a NES (tCherry–NES) and thus marked the cytoplasm. Both translation factors show nuclear accumulation only in *pdr6*Δ cells. White arrows point to nuclei in the merged images. Bars, 5 µm.

Table S1. **Plasmids used in this study**

Number	Construct	Purpose	Used in
pAV0076	bdSUMO-HygB-bdSUMO(G96A, G97A)	Protease-specificity sensor	Fig. 1
pAV0160	bdSUMO(G96A, G97A)-FLFVQ-HygB-bdSUMO-ssrA	Protease-specificity sensor	Fig. 1
pAV0159	bdSUMO-FLFVQ-HygB-bdSUMO-ssrA	Protease-specificity sensor	Fig. 1
pAV0161	bdSUMO-FLFVQ-HygB-bdSUMO(G96A, G97A)-ssrA	Protease-specificity sensor	Fig. 1
pAV0162	bdSUMO(G96A, G97A)-FLFVQ-HygB-bdSUMO(G96A, G97A)-ssrA	Protease-specificity sensor	Fig. 1
pSF2507	H14-Tev-bdSENP1 (RBS m-22)	Protease co-expression plasmid	Fig. 1
pAV0179	H14-Tev-SUMOstar protease (RBS m-22)	Protease co-expression plasmid	Fig. 2
pAV0171	bdSUMO-FLFVQ-HygB-SUMOstar-ssrA	Protease-specificity sensor	Fig. 2
pAV0625	scSUMO-FLFVQ-HygB-bdSUMO^{Eu1}-ssrA	Protease-specificity sensor	Fig. 2
pAV0608	H14-hsSUMO2-MBP	Bacterial expression	Fig. 3
pAV0231	H14-scSUMO-MBP-FlagTag	Bacterial expression	Figs. 3, S2, and S3
pAV0353	H14-SUMO^{Eu0}-MBP-FlagTag	Bacterial expression	Fig. 3
pAV0279	H14-SUMO^{Eu1}-MBP-FlagTag	Bacterial expression	Figs. 2, 3, S2, and S3
pAV0280	H14-SUMO^{Eu8}-MBP-FlagTag	Bacterial expression	Fig. 3
pAV0277	H14-SUMO^{Eu10}-MBP-FlagTag	Bacterial expression	Fig. 3
pAV0700	H14-SUMO^{Eu11}-MBP-FlagTag	Bacterial expression	Fig. 3
pAV0698	H14-SUMO^{Eu12}-MBP-FlagTag	Bacterial expression	Fig. 3
pAV0701	H14-SUMO^{Eu13}-MBP-FlagTag	Bacterial expression	Fig. 3
pAV0696	H14-SUMO^{Eu14}-MBP-FlagTag	Bacterial expression	Fig. 3
pAV0697	H14-SUMO^{Eu15}-MBP-FlagTag	Bacterial expression	Fig. 3
pSF1877	H14-Tev-scUlp1	Bacterial expression	Figs. 2, 3, and S3
pSF1878	H14-Tev-SUMOstar protease	Bacterial expression	Figs. 2, 3, and S2
pAV0605	H14-MBP-bdNEDD8-hsSENP2	Bacterial expression	Figs. 2 and 3
pAV0286	H14-Tev-SENP1^{EuB}	Bacterial expression	Figs. 3, 4, 5, 6, S2, S3, and S4
pAV0678	H14-Tev-SENP^{EuG}	Bacterial expression	Fig. 3
pAV0679	H14-Tev-SENP^{EuH}	Bacterial expression	Fig. 3
pAV0680	H14-Tev-SENP^{Eui}	Bacterial expression	Fig. 3
pAV0681	H14-Tev-SENP^{EuJ}	Bacterial expression	Fig. 3
pAV0682	H14-Tev-SENP^{EuK}	Bacterial expression	Fig. 3
pAV0233	H14-bdSUMO-MBP-FlagTag	Bacterial expression	Figs. 2, S2, and S3
pAV0352	H14-bdSUMO(T60K)-MBP-FlagTag	Bacterial expression	Fig. 2
pAV0354	H14-bdSUMO(Q75R)-MBP-FlagTag	Bacterial expression	Fig. 2
pAV0356	H14-bdSUMO(T60K; Q75R)-MBP-FlagTag	Bacterial expression	Fig. 2
pAV0355	H14-bdSUMO(T60K; D67K)-MBP-FlagTag	Bacterial expression	Fig. 2
pSF2554	ZZ-scSUMO-YFP	Yeast expression	Fig. S2
pAV0329	ZZ-SUMOstar-YFP	Yeast expression	Fig. S2
pSF2565	ZZ-bdSUMO-YFP	Yeast expression	Fig. S2
pAV0317	ZZ-SUMO^{Eu1}-YFP	Yeast expression	Fig. S2
pAV0313	ZZ-bdSENP1	Yeast expression	Fig. S2
pAV0316	ZZ-SUMOstar protease	Yeast expression	Fig. S2
pAV0315	ZZ-scUlp1	Yeast expression	Fig. S2
pAV0320	ZZ-SENP1^{EuB}	Yeast expression	Fig. S2
pAV0319	ZZ-bdSENP1(C440S)	Yeast expression	Fig. S2
pAV0331	H14-SUMO^{Eu1}-Nb (‘enhancer’)	Yeast expression	Fig. S2

Table S1. **Plasmids used in this study (*Continued*)**

Number	Construct	Purpose	Used in
pAV0232	H14-scSUMOstar-MBP-FlagTag	Bacterial expression	Fig. 3
pAV0719	H14-eGFP-SUMO^{Eu1}-hsActinAP	HEK cells expression	Fig. S3
pAV0346	ED-SUMO^{Eu1}-H12-Pdr6	Bacterial expression	Figs. 4, 5, 6, and S4
pMA0239	H14-bdNEDD8-Pdr6	Bacterial expression	Figs. 6 and S4
pSF1004	H14-Tev-Gsp1-GTP (Q71L ΔC-terminus mutant)	Bacterial expression	Figs. 4, 5, 6, and S4
pAV0363	H14-bdNEDD8-eIF5A	Bacterial expression	Fig. 5
pAV0566	H14-ZZ-bdNEDD8-Ubc9	Bacterial expression	Fig. 5
pAV749	H14-ZZ-bdNEDD8-Pil1(39-267)	Bacterial expression	Fig. S4
pAV750	H14-ZZ-bdNEDD8-Pil1	Bacterial expression	Fig. S4
pAV0767	H14-ZZ-bdNEDD8-Lsp1	Bacterial expression	Fig. S4
pAV0515	tCherry-NES	Yeast expression	Figs. 5 and S5
pAV0514	NLS-tCherry	Yeast expression	Fig. 6
pAV0498	ED-SUMO^{Eu1}-H12-eIF5A	Bacterial expression	Fig. 6
pDG2506	His14-ScSUMO-ZpA963 dimer-spacer-Cys	Bacterial expression	Figs. 4, 5, 6, and S4

Maps, sequences, and actual plasmids are available on request. Plasmids of general interest will be distributed through AddGene.

References

Joel, P.B., P.M. Fagnant, and K.M. Trybus. 2004. Expression of a nonpolymerizable actin mutant in Sf9 cells. *Biochemistry*. 43:11554–11559. https://doi.org/10.1021/bi048899a

Mossessova, E., and C.D. Lima. 2000. Ulp1-SUMO crystal structure and genetic analysis reveal conserved interactions and a regulatory element essential for cell growth in yeast. *Mol. Cell.* 5:865–876. https://doi.org/10.1016/S1097-2765(00)80326-3

Reverter, D., and C.D. Lima. 2004. A basis for SUMO protease specificity provided by analysis of human Senp2 and a Senp2-SUMO complex. *Structure*. 12:1519–1531. https://doi.org/10.1016/j.str.2004.05.023
